# A multi-stage feature alignment framework with cross-modality collaborative fusion for visible-infrared person re-identification

**DOI:** 10.1038/s41598-026-50904-5

**Published:** 2026-04-28

**Authors:** Guangjie Liu, Wanping Yang, Yingwen Zhang, Xiutao Zhao

**Affiliations:** College of Computer Science and Technology, Changchun Normal University, 677 Changji North Road, Changchun, 130031 China

**Keywords:** Visible-infrared person re-identification, Modality difference optimization, Multi-stage hybrid-modality alignment, Collaborative preprocessing, Computational biology and bioinformatics, Engineering, Mathematics and computing

## Abstract

Efficient implementation of Visible-Infrared Person Re-identification (VI-ReID) is important for intelligent transportation and surveillance systems, where the primary challenge lies in the semantic discrepancy between visible and infrared modalities. Although existing methods have made progress in mitigating modality discrepancies, single-stage feature alignment remains prone to semantic shifts. We argue that hierarchical transitions in the feature alignment process can alleviate this issue. Based on this observation, we propose a Multi-Stage Feature Alignment and Cross-Modality Collaborative Fusion (MS-CF) framework, which performs multi-dimensional modality discrepancy optimization and suppresses redundant information, thereby ensuring cross-modality consistency and intra-modality stability. The MS-CF framework consists of three modules. The Dual-Path Cross-Layer Attention (DCA) module enhances semantic and structural representations of intermediate features. The Balanced Feature Normalization (BFN) module improves feature distribution consistency and discriminability by incorporating modality constraints and feature sparsification. The Multi-Stage Hybrid-Modality Alignment (MS-HMA) strategy applies collaborative constraints to hybrid modalities after initial single-modality alignment, enabling coarse-to-fine semantic convergence. Extensive experiments on the SYSU-MM01 and RegDB datasets demonstrate that the MS-CF framework outperforms state-of-the-art methods. In particular, under the indoor-search setting of SYSU-MM01, MS-CF achieves improvements of 5.75% in Rank-1 accuracy and 3.97% in mAP, validating its effectiveness in enhancing robustness and recognition performance.

## Introduction

 Person re-identification (ReID) aims to match images of the same individual across different cameras and viewpoints using deep learning techniques^1^. Its primary objective is to extract identity-invariant representations and optimize the feature space through metric learning. Traditional ReID models mainly rely on appearance cues, such as clothing color and texture, for discrimination^2,3^. However, illumination variations between indoor and outdoor cameras, especially under low-light conditions, RGB images often suffer from blur and distortion, resulting in reduced informativeness and degraded performance. In contrast, infrared (IR) cameras capture thermal radiation, enabling stable imaging in dark environments but lacking color and texture information^4^. This creates a substantial modality gap between infrared (IR) and visible spectrum (RGB) data^5^. To address this issue, VI-ReID focuses on aligning cross-modality features and modeling identity consistency across modalities, thereby improving recognition performance under challenging lighting conditions.

VI-ReID further focuses on the reduction of modal differences on the basis of traditional ReID, which has potential influence in the field of monitoring and security. VI-ReID faces significant challenges due to modality discrepancies, including intra-modality variations caused by viewpoint changes and inter-modality differences arising from distinct sensing mechanisms^6^. These discrepancies increase the difficulty of aligning infrared (IR) and visible (RGB) features, resulting in identity inconsistency within the feature space and ultimately leading to person matching failures^7^. Existing feature alignment methods can be broadly categorized into explicit alignment and implicit alignment. Explicit alignment typically relies on modality transformation or mapping techniques, such as adversarial training, image generation^8^, or distribution matching^9^, to unify multi-modality features in a shared space, directly optimizing cross-modality consistency. In contrast, implicit alignment emphasizes learning shared feature spaces^10^ or employing feature fusion strategies^11^, encouraging the extraction of modality-invariant discriminative features through network architecture, loss functions^12^, or attention mechanisms^13^. However, explicit alignment methods often rely heavily on the quality of generated images or feature mappings, which may introduce noise and artifacts. Implicit alignment, while improving modality invariance, may overlook modality-specific characteristics, resulting in a loss of discriminative information. Therefore, achieving effective cross-modality feature alignment while preserving identity-discriminative cues remains a critical research challenge. In particular, without a natural modality bridge, directly measuring feature distances across modalities frequently induces semantic shifts, leading to severe deviations in the feature-space representations of the same identity^14^. Hence, designing a reasonable modality bridging mechanism that enhances discriminability, preserves modality-specific information, and suppresses redundant cues is of considerable research importance.

To achieve smooth cross-modality feature alignment, we propose a deep learning framework integrating a collaborative preprocessing mechanism with a multi-stage hybrid-modality alignment module, denoted as the MS-CF framework. The collaborative preprocessing mechanism progressively optimizes feature representations from three aspects, including modality bridging, semantic enhancement, and representation stability, by constructing a structured module chain to enhance cross-modality consistency. We introduce the grayscale modality as an intermediate bridge to facilitate a natural transition between modalities and mitigate input distribution bias^15^. The grayscale modality preserves structural information from visible images while avoiding pseudo-differences caused by color interference. A Dual-Path Cross-Layer Attention (DCA) mechanism reconstructs the structural fusion paradigm to enhance cross-modality generalization while preserving intra-modality details. This mechanism is embedded in intermediate layers to model critical regions during the transition from texture to semantic representations. For expression stability, Balanced Feature Normalization (BFN) incorporates L2 regularization and the ReLU activation function into conventional affine normalization. L2 regularization suppresses excessively large weights, while ReLU enhances the nonlinear representational capacity of features. Although this combination is rarely employed in standard normalization processes, it demonstrates advantages of strong expressiveness and stable amplitude in tasks targeting the reduction of modality discrepancies. Building upon collaborative preprocessing, we further propose a Multi-Stage Hybrid-Modality Alignment (MS-HMA) mechanism. Unlike conventional approaches that align modalities independently, this mechanism leverages fused multi-modality representations as new alignment units to perform semantic reconstruction and enforce collaborative constraints. The overall design is progressive and lightweight, avoiding the accumulation of additional parameters. Instead, it employs a similarity-guided feature reconstruction strategy to fully exploit complementary information across modalities.

The main contributions of this work are summarized as follows:

1. We propose the MS-CF framework, which progressively optimizes modality discrepancies from three dimensions: semantic enhancement, expression stability, and feature unification.
2. We design DCA, BFN, and MS-HMA to leverage the stage-specific characteristics during training, which helps improve the consistency of cross-modality representations and progressively achieve coarse-to-fine cross-modality alignment.
3. Extensive experimental results demonstrate that the proposed mechanism outperforms existing mainstream methods on two large-scale VI-ReID benchmark datasets.

## Related work

### Visible-infrared person re-identification

The core challenge of VI-ReID lies in the modality discrepancies introduced by cross-modality recognition. These discrepancies cause significant shifts in the feature space distributions between visible and infrared images, which severely hinder cross-modality matching performance. To alleviate this issue, researchers have proposed various approaches. For example, one study^7^ bridges the modality gap by implementing modality compensation in the feature space, generating missing modality-specific features from modality-shared features. Combined with feature decomposition and fusion mechanisms, it effectively separates and integrates modality-specific and modality-shared information, progressively reducing distribution differences in the feature space. Another study^16^ learns independent proxies for each modality and employs a diverse proxy memory bank to perform uni-directional metric learning and memory-augmented matching. This design improves cross-modality associations and robustness under modality imbalance. Furthermore, to further reduce modality discrepancies, researchers have adopted different mechanisms to remove modality-sensitive features and reinforce high-level discriminative semantic features shared across modalities. For instance, one work^17^ jointly optimizes attribute-guided fine-grained feature extraction with modality consistency constraints to create a more compact feature space. Another study^18^ employs orthogonal decomposition and shape-erasing feature learning paradigms to break the model’s dependence on shape information, explicitly guiding it to learn diverse modality-shared semantic features. Recent works also emphasize that auxiliary feature modeling can improve this issue. One study^19^ proposes modeling based on high-order structural information. It uses a whitening hypergraph network to capture complex local dependencies and applies graph attention to align features across modalities and scales, generating reliable intermediate features to boost matching performance. Another study^20^ introduces a dual-stream framework for information purification and multi-level knowledge distillation. The information purification module removes style differences, while the dual-stream architecture separates feature types. By incorporating feature-level and semantic-level knowledge distillation along with modality discrepancy reduction losses, this method alleviates the underutilization of discriminative information in modality-specific features. It also reduces the negative impact of modality discrepancies on direct alignment.

However, most existing methods primarily focus on modeling in the high-level representation space, while neglecting the persistent modality shifts within hierarchical features. These modality discrepancies do not exist in isolation but propagate and accumulate across network layers, becoming the root cause of inconsistencies in high-level features. Therefore, we propose a collaborative preprocessing mechanism to systematically address modality-sensitive signals.

### Feature alignment

Feature alignment aims to map heterogeneous modality-specific features into a shared semantic space to reduce cross-modality distribution gaps. Feature alignment methods are generally divided into explicit alignment and implicit alignment. Explicit alignment often employs adversarial training to map one modality form to another, or maps both modalities into a unified space. Distance metric learning is also used to directly constrain cross-modality feature distances. For example, one study^8^ integrates generative adversarial learning with a teacher-student guided multi-level feature alignment framework to achieve cross-modality feature mapping through collaborative alignment in both image and feature spaces. Another study^9^ proposes a walk-based fine-grained feature alignment mechanism that involves semantic mask generation, error correction, and pixel-level contrastive learning. This mechanism alleviates modality discrepancies at the shallow spatial level.

A recent study^21^ explicitly reduces the modality gap between visible and infrared images by designing a modality alignment augmentation method with weighted grayscale, cross-channel CutMix, and spectrum jitter, combined with a cross-modality retrieval loss based on ranking lists, aligning features at both the image and feature levels. Implicit alignment leverages shared network weights, shared attention mechanisms, and other strategies to enhance cross-modality information fusion. For instance, one study^22^ forms dual implicit feature alignment from input to feature representations by combining pixel-level modality-adaptive mixing with feature-level modality-adaptive convolution decomposition. This promotes the formation of a continuous modality-invariant latent space. Another study^23^ introduces a semantic-consistent part-level enhancement method that constructs cross-modality positive and negative pairs in the feature space. It jointly employs entropy-aware filtering strategies and contrastive regularization to effectively achieve cross-modality feature alignment. In addition, study^24^ proposes a more robust heterogeneous feature learning approach that coordinates alignment at the sample relationship, statistical distribution, and dynamic normalization levels, balancing modality specificity and discriminative consistency. This method effectively mitigates feature degradation caused by modality discrepancies in implicit feature alignment.

In addition, other visual tasks have also explored cross-modality feature alignment in depth. For example, in the RGB-D salient object detection task, a study^25^ employs a Conformer dual-stream structure to separately extract RGB and depth features. At shallow layers, the SCLR module learns modality-correlated representations between texture and structure, while at deeper layers, the DCLR module models cross-modal semantic correlations. A feature coupling unit is also used to fuse the CNN and Transformer streams, constructing robust cross-modal salient object representations. In pedestrian attribute recognition, a study^26^ proposed a dual-branch Transformer network that integrates local and global features through a cross-fusion mechanism to alleviate feature inconsistency. Building on this, subsequent work^27^ extended the approach to cross-modal attribute-based pedestrian retrieval by designing a unified network that simultaneously handles attribute recognition and retrieval, and aligns visual features with language features generated from pseudo-text via a cross-modal Transformer, enabling effective feature fusion and improved cross-modal matching. In text-guided video action understanding, RefAtomNet^28^ first proposed a cross-stream attention method to recognize atomic actions of target individuals. Building on this, RefAtomNet++^29^ extends the approach by employing multi-hierarchical semantic-aligned cross-attention combined with multi-trajectory Mamba modeling to dynamically aggregate visual tokens at the keyword, scene-attribute, and holistic-sentence levels, enabling effective cross-modal alignment between text and video and improving fine-grained action recognition accuracy. Recently, HopaDIFF^30^ introduced the textual reference-guided human action segmentation task and proposed a holistic-partial aware Fourier conditioned diffusion framework. It leverages a cross-input gate attentional xLSTM to enhance long-range reasoning and incorporates a Fourier condition for fine-grained control, effectively aligning textual descriptions with target visual features.

Existing methods have largely alleviated the overall distribution discrepancies between modalities. However, these methods often regard cross-modality alignment as a single-level or one-shot process, overlooking the hierarchical and multi-stage complex heterogeneous information coupling between different modalities. Motivated by this observation, we propose a multi-stage hybrid-modality alignment method that progressively aligns single-modality and hybrid-modality feature spaces, effectively enhancing cross-modality ReID performance.

## Methodology

**Fig. 1:**
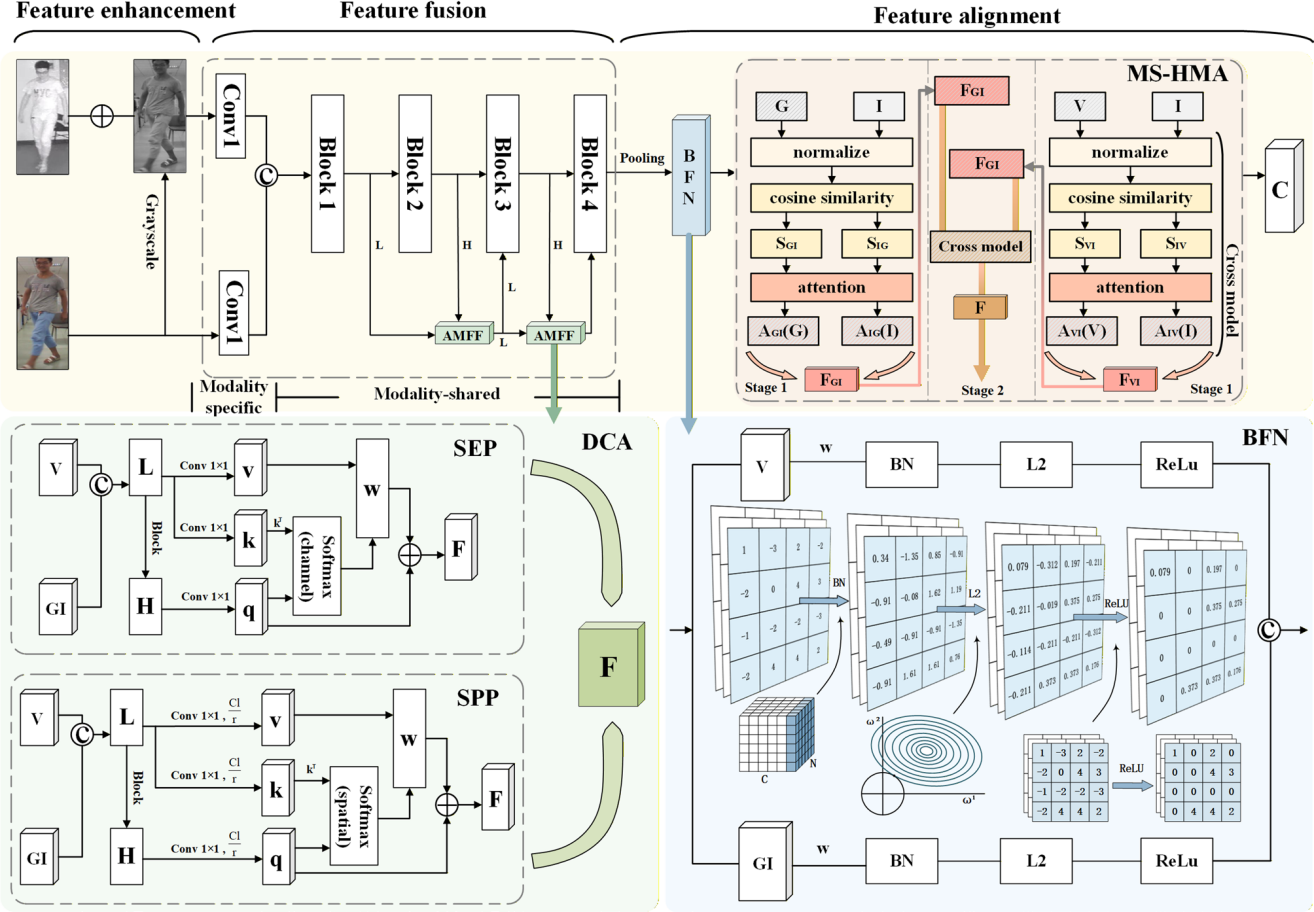
The MS-CF framework consists of three core modules: DCA, BFN, and MS-HMA. Built upon a ResNet-50 backbone, the model adopts a dual-stream architecture. Modality-specific layers extract shallow features for each modality, while modality-shared layers capture deep features with shared weight parameters. DCA captures global contextual relationships, while BFN and MS-HMA enhance feature stability and modality consistency, respectively, thereby improving overall feature representation.

The MS-CF framework adopts a unified progressive design that tightly couples the collaborative preprocessing mechanism with the multi-stage hybrid-modality alignment module, as illustrated in Fig. 1. The overall pipeline follows the principle of fusion first, alignment later, and gradual convergence. Through collaborative preprocessing, cross-modality feature distributions are progressively stabilized. On this basis, multi-stage hybrid-modality alignment is introduced to achieve gradual semantic consistency among visible, infrared, and grayscale modalities. This approach effectively mitigates feature drift caused by modality discrepancies. It is worth emphasizing that DCA and BFN alleviate semantic-structural inconsistencies and distribution fluctuations, providing stable features for the stage-wise alignment in MS-HMA.

Specifically, the collaborative preprocessing mechanism consists of DCA and BFN. DCA is embedded in the intermediate layers of the backbone network, where positional information and a dual-path attention mechanism are introduced to enhance cross-modality semantic-structural complementarity. BFN is applied at the feature aggregation stage to normalize feature distributions and improve representation stability. Building upon this foundation, MS-HMA adopts a two-stage sequential alignment strategy. Visible-infrared and grayscale-infrared pairs are first aligned in a single-modality manner. The fused features are then re-aligned to achieve more comprehensive hybrid-modality semantic convergence. These components are functionally distinct yet sequentially connected, collectively forming a progressive cross-modality alignment pipeline driven by collaborative fusion.

Formally, we represent the visible image, infrared image, and grayscale image in the dataset as,

1 $$V = \left\{ {X_{{Vi}} } \right\}_{{i = 1}} ,\:I = \left\{ {X_{{Ii}} } \right\}_{{i = 1}} ,\:G = \left\{ {X_{{Gi}} } \right\}_{{i = 1}}$$

and then concatenate G and I as,

2 $$\:GI={\left\{{X}_{GIi}|{X}_{GIi}\in\:I\cup\:G\right\}}_{i=1}$$

and denote the image in the mini-batch sampling as,

3 $$\:X={\left\{{X}_{i}|{X}_{i}\in\:V\cup\:GI\right\}}_{i=1}$$

Equations (2) and (3) are only used to define the data sets and do not involve any specific feature alignment operations. Due to the imbalance in the number of RGB and IR images in the SYSU-MM01 dataset (with more RGB images than IR images), mini-batches are constructed based on person identities during training. Specifically, an index mapping between identities and their corresponding samples is first established according to the identity labels of the RGB and IR images. At the beginning of each training epoch, samples are randomly selected according to identities to construct the mini-batch. In this way, each batch contains samples from both RGB and IR modalities, which helps alleviate the influence of modality imbalance caused by the different numbers of samples in the two modalities during training. The model adopts a multi-image input structure during training. Each training sample contains three images: a visible (RGB) image, a grayscale image converted from the RGB image, and an infrared (IR) image. The grayscale image is generated by converting the RGB image into a single-channel grayscale representation and then replicating it into three channels. During training, the RGB image and its grayscale counterpart are first concatenated along the batch dimension and then separated at the beginning of the forward pass. The RGB image is fed into the visible branch to extract visible features, while the grayscale and infrared images are concatenated along the batch dimension and processed by the thermal branch to extract infrared-related features. The input image X is fed into two feature extraction branches (Conv 1) to separately capture modality-specific representations. Subsequently, the intermediate outputs of both branches are concatenated and fed into a modality-shared network, which progressively extracts deeper shared features from Block1 to Block4. To enhance semantic consistency across layers, DCA modules are introduced after Block2 and Block3, receiving low-level features L and high-level features H, respectively. Through a structurally parallel design, these modules guide the complementary reconstruction of semantic and structural information, mitigating redundancy in the attention pathways. The aggregated features are pooled and subsequently normalized by the BFN module to improve stability. The BFN module processes the features by sequentially applying Batch Normalization (BN), L2 regularization, and ReLU activation, and then concatenates the outputs to produce the final normalized representation. During the alignment stage, preliminary intra-modality alignments are initially performed based on G-I and V-I pairs. Then, the two sets of aligned features undergo hybrid-modality fusion, combining mutual feature reconstruction and alignment constraints. This forms a unified discriminative representation, which is fed into the classifier (C). Through this staged transition, feature drift caused by modality discrepancies is effectively suppressed, thereby enhancing cross-modality matching accuracy and robustness.

### Dual-path cross-layer attention

As a collaborative fusion module bridging the early and intermediate stages of the MS-CF framework, the DCA module is designed to facilitate hierarchical feature interaction and cross-modality semantic-structural complementarity. Traditional self-attention mechanisms exhibit global modeling capabilities. However, their single-path design struggles to reconcile the multi-level requirements of semantic consistency and local structural recognition. This often results in conflicts between semantic and structural representations during cross-modality alignment. To address this limitation, we propose the DCA mechanism as a core module for hierarchical feature alignment, designed to enhance fine-grained complementary interactions across modalities.

In traditional multi-layer attention mechanisms, subsequent modules are usually directly stacked on the outputs of previous layers, leading to tightly coupled pathways, redundant information flow, and amplified noise. Compared to traditional attention fusion mechanisms such as CBAM^31^, our DCA redefines attention fusion based on the principle of functional decoupling, achieving dual-path collaborative complementarity from the perspective of functional specialization. We observe that conventional fusion methods often over-rely on already processed features, resulting in information loss and noise accumulation. To address this limitation, DCA employs a strategy termed dual independent learning of initial features. In this strategy, the two paths concentrate on different levels of representation, thereby preserving original feature information while effectively mitigating noise interference. Additionally, we systematically investigate the optimal insertion timing and find that, following preliminary feature refinement, intermediate layers act as critical repositories for high-quality features. Introducing the dual-path cross-layer attention mechanism at this stage fully exploits its advantages, significantly improving model performance. Specifically, SEP utilizes high-level features X_h as queries and low-level features X_l as keys and values to construct an asymmetric non-local relational graph, thereby capturing long-range semantic dependencies. The formulation is expressed as,

4 $$\:{A}_{SEP}=\frac{1}{{N}_{l}}\theta\:\left({X}_{h}\right)\cdot\:{\phi\:\left({X}_{l}\right)}^{T}$$

5 $$\:{Z}_{SEP}={W}_{SEP}\left(reshape\left({A}_{SEP}\cdot\:g\left({X}_{l}\right)\right)\right)+{X}_{h}$$

where $\:{A}_{SEP}$​ denotes the semantic similarity matrix between high-level and low-level features, and $\:\frac{1}{{N}_{l}}$ is a normalization factor to prevent energy explosion. The feature tensors after the 1 × 1 convolutional mappings are respectively,

6 $$\mathrm{g}=\mathrm{g}\left({X}_{l}\right)\in {R}^{B\times {C}_{l}\times {H}_{l}\times {W}_{l}}, \theta =\theta \left({X}_{h}\right)\in {R}^{B\times {C}_{l}\times {H}_{h}\times {W}_{h}}, \phi =\phi \left({X}_{l}\right)\in {R}^{B\times {C}_{l}\times {H}_{l}\times {W}_{l}}.$$

SPP guides attention weights through channel reduction and spatial convolution, enhancing positional awareness and thereby aligning local textures and fine-grained structural details. The formulation is expressed as,

7 $${A}_{SPP}=\frac{1}{{N}_{l}}\theta {\left({X}_{h}\right)}^{T}\cdot\phi \left({X}_{l}\right)$$

8 $${Z}_{SPP}={W}_{SPP}\left(reshape\left({A}_{SPP}\cdot g{\left({X}_{l}\right)}^{T}\right)\right)+{X}_{h}$$

where ${A}_{SPP}$ represents the spatial attention weight matrix in the SPP path, computed from the dimension-reduced high-level and low-level features. It is used to guide the weighted aggregation of low-level features. In this path, the channel dimension is reduced to $\:\frac{{C}_{l}}{r}$ (where r is the channel reduction ratio).

9 $${\mathrm{g}} = {\mathrm{g}}\left( {X_{l} } \right) \in R^{{B \times \frac{{C_{l} }}{r} \times H_{l} \times W_{l} }} ,\theta = \theta \left( {X_{h} } \right) \in R^{{B \times \frac{{C_{l} }}{r} \times H_{h} \times W_{h} }} ,\phi = \phi \left( {X_{l} } \right) \in R^{{B \times \frac{{C_{l} }}{r} \times H_{l} \times W_{l} }}.$$

Then, the two outputs are fused to obtain,

10 $$\:Z=\alpha\:\cdot\:{Z}_{SEP}+{(1-\alpha\:)\cdot\:Z}_{SPP}$$

This fusion strategy performs weighted integration at the path level, preserving the discriminability of semantic representations while enhancing the precision of structural information, thereby embodying an innovative decoupled-collaborative paradigm for attention modeling. Unlike methods such as DDAG^17^, which fuse intra- and inter-modality attention within a single path to jointly model all dependencies, we decouple semantic abstraction and structural compensation into two independent modeling paths, effectively avoiding interference during feature interaction. Further systematic experiments demonstrate that inserting DCA into shallow layers increases the model’s susceptibility to noise, whereas placing it in deeper layers results in excessively abstract semantic representations with insufficient structural details. Conversely, positioning DCA in the middle layers of the backbone network, where transitions between semantic and structural information occur, enables its dual-path mechanism to maximize collaborative effectiveness. These findings suggest that attention modeling should prioritize semantic compatibility rather than rely solely on hierarchical stacking, providing a structural re-evaluation of conventional attention usage strategies.

### Balanced feature normalization

After DCA completes the intermediate-stage fusion, the BFN module is introduced to further regularize the cross-modality feature distributions and stabilize the representations prior to explicit matching. The BFN module is designed to address common issues of training instability and representational bias in multi-modality feature alignment. In Batch Normalization (BN), the affine transformation parameters, scaling (γ) and shifting (β), are generally learned directly from data without explicit regularization constraints. However, in multi-modal tasks, unconstrained learning may amplify distributional discrepancies between modalities. This can lead to statistical shifts, training instability, and overfitting during alignment. To mitigate this issue, we introduce, for the first time, L2 regularization on the affine parameters within BN. Specifically, for the input feature X, which represents the fused output from the backbone network combined with the DCA module, the corresponding affine-transformed representation is given by

11 $$\:Y=X\odot\:\gamma\:+\beta\:$$

Here, $\:\odot\:$ denotes element-wise multiplication. To suppress distribution drift caused by excessively large affine parameters, we introduce the following regularization term,

12 $${\mathcal{L}}_{{L2}} = \lambda \left( {\left\| \gamma \right\|_{2}^{2} + \left\| \beta \right\|_{2}^{2} } \right)$$

Here, $\:\lambda\:$ is the regularization coefficient. This regularization term encourages the affine parameters of multimodal features to remain reasonable and independent, adapting to complex distributions. To further improve the nonlinear representation capability of the features, we introduce a ReLU activation following the BN affine transformation,

13 $$\:\stackrel{\sim}{Y}=ReLU\left(Y\right)$$

ReLU not only substantially enhances the discriminative power of representations in cross-modality alignment but also partially offsets the representational compression induced by L2 regularization. The combination of these two components constitutes a unified normalization structure, balancing numerical stability with nonlinear discriminative capacity.

It is important to emphasize that the BFN module is a task-specific optimization mechanism explicitly designed to address the heterogeneous nature of features in VI-ReID. Empirical studies show that using L2 regularization alone can suppress parameter oscillations but tends to cause excessive feature smoothing. In contrast, employing ReLU alone enhances discriminative power while inducing noticeable fluctuations in training loss. By integrating L2 regularization and ReLU in a coordinated manner, the BFN module attains an optimal balance between training stability and feature expressiveness (see ablation study in Table 5 for details).

### Multi-stage hybrid modality alignment

Based on the progressively fused and normalized features produced by DCA and BFN, we propose a Multi-Stage Hybrid Modality Alignment (MS-HMA) module to explicitly model the cross-modality semantic alignment process. Extending the traditional single-modality alignment paradigm, MS-HMA performs pairwise cross-modality soft-attention alignment among the visible, infrared, and grayscale modalities. By combining normalized cosine similarity with a bidirectional attention mechanism, we achieve feature reweighting. This is formally expressed as,

14 $$\:\widehat{F}_{V} = \frac{{F_{V} }}{{\left\| {F_{V} } \right\|_{2} }},\widehat{F}_{I} = \frac{{F_{I} }}{{\left\| {F_{I} } \right\|_{2} }}$$

where $\:{F}_{V}\in\:{R}^{N\times\:C}$ denotes the infrared modality features, and $\:{F}_{I}\in\:{R}^{N\times\:C}$ denotes the visible modality features. Here, all features $\:{F}_{*}$ refer to the outputs of the backbone network after normalization and activation.

15 $$\:{S}_{V\to\:I}={\widehat{F}}_{V}{{\widehat{F}}_{I}}^{T}\:,\:{S}_{I\to\:V}={{S}_{V\to\:I}}^{T}$$

16 $$A_{{V \to I}} = softmax\left( {S_{{V \to I}} /\tau } \right),A_{{I \to V}} = softmax\left( {S_{{I \to V}} /\tau } \right)$$

17 $$\:{F}_{V}^{{\prime\:}}={A}_{V\to\:I}{F}_{I}\:,\:{F}_{I}^{{\prime\:}}={A}_{I\to\:V}{F}_{V}$$

However, we observe that traditional single-modality alignment methods generally overlook the hierarchical differences in semantic-structural cross-modality interactions (e.g.,^22^). This limitation is particularly pronounced with the introduction of the grayscale modality, which naturally aligns with the infrared modality in terms of texture and contrast representation. Naively concatenating these modalities often induces redundancy amplification and semantic drift. Motivated by this observation, we propose the MS-HMA module, which possesses progressive semantic convergence capability. The core concept is to refine the conventional one-shot coarse alignment into a two-stage hierarchical modeling process. The first stage emphasizes intra-modality collaborative convergence, whereas the second stage establishes a fine-grained fusion path to facilitate semantic interaction across modalities. In contrast to existing methods that typically perform coarse-grained alignment only at the final feature level, the MS-HMA module sequentially models and aligns different modalities and their fused representations in a stage-wise manner. This approach results in gradual feature space convergence and enhanced semantic consistency, significantly improving the inherent bridging capability between modalities. Specifically, in the first stage, alignment is conducted along two sub-paths: visible-infrared and grayscale-infrared. These sub-paths are aligned using soft attention mechanisms and subsequently concatenated to produce two intermediate mixed-modality representations. In the second stage, rather than merely fusing these features, another round of alignment is performed using a newly designed cross-modality soft attention module to refine the fused representations from both sub-paths. This process not only mitigates information redundancy but also explicitly models semantic collaboration and complementary differences among the three modalities. Compared with traditional approaches, our method achieves more precise alignment at both structural and semantic levels.

Additionally, to ensure representation consistency and training stability across different fusion stages, we introduce a consistency regularization term based on the Mean Squared Error (MSE). This regularization term minimizes the representational discrepancy between outputs from different fusion paths, thereby effectively mitigating feature drift and modality conflicts. Specifically, we impose a MSE constraint between the final fused feature $\:{v}_{sft}$ and $\:{v}_{sft\_RI}$ to enhance their semantic consistency. Here, $\:{v}_{sft}$ denotes the output from the visible-infrared single-modality alignment path, while $\:{v}_{sft\_RI}$ is derived from the grayscale-infrared single-modality alignment. This consistency regularization encourages convergence between the two complementary alignment branches and ensures stable fusion in the subsequent hybrid-modality integration stage.

18 $$\:{\mathcal{L}}_{mse}=MSE\left({v}_{sft}\:,{v}_{sft\_VI}\right)$$

To further enhance the model’s robustness and alignment accuracy, we adopt a multi-objective optimization strategy during training by jointly employing cross-entropy loss $\:{\mathcal{L}}_{ce}$, triplet loss $\:{\mathcal{L}}_{tri}$, second-order distribution alignment loss $\:{\mathcal{L}}_{aff}$, and consistency loss $\:{\mathcal{L}}_{mse}$,

19 $$\:{\mathcal{L}}_{total}={\mathcal{L}}_{ce}+{\mathcal{L}}_{tri}+{\mathcal{L}}_{aff}+{\lambda\:\cdot\:\mathcal{L}}_{mse}$$

During the inference stage, to address potential residual noise within the fused features, we introduce a lightweight similarity-based filtering and mean reconstruction mechanism. This strategy retains only the feature components most semantically relevant to the query image, thereby further enhancing the accuracy and stability of cross-modality recognition. This process is formally defined as,

20 $$\:{q}_{f}^{new}=Concat\left(TopKAvg\left({\widehat{q}}_{i}\:,\widehat{g}\right)\right)\:,\:{g}_{f}^{new}=Concat\left(TopKAvg\left({\widehat{g}}_{i}\:,\widehat{g}\right)\right)$$

### End-to-end workflow of MS-CF

Given an input image, the model first extracts modality-specific shallow features and progressively builds deep representations through a shared backbone network. DCA is introduced in the intermediate layers to model cross-modality interactions and enhance semantic-structural complementarity. It is then followed by BFN, which normalizes the aggregated features to stabilize cross-modality distributions. Building on these refined representations, MS-HMA performs stage-wise hybrid-modality alignment to produce the final discriminative embeddings for cross-modality matching.

## Experiments

### Datasets and experimental settings

#### Datasets

We conducted a comprehensive evaluation of our proposed method on two well-known publicly available VI-ReID datasets: SYSU-MM01^32^ and RegDB^33^. SYSU-MM01 was collected on the campus of Sun Yat-sen University using four RGB cameras and two near-infrared (IR) cameras, comprising annotations for 491 different identities. This dataset offers two experimental settings: All-Search and Indoor-Search. The training set contains 19,659 RGB images and 12,792 IR images, covering 395 identities. The test set includes 96 identities. RegDB is a smaller-scale dataset collected using one visible camera and one thermal infrared camera. It contains 8,240 images of 412 pedestrians, with 10 visible and 10 infrared images per person. The dataset ensures gender diversity, including 254 female and 158 male subjects. Each person is captured from both front and back views, with 156 front-view and 256 back-view identities. Among them, 206 identities are allocated for training, and the other 206 for testing.

#### Evaluation metrics

Cumulative Match Characteristic (CMC) and mean Average Precision (mAP) are used as the main evaluation indicators. CMC^34^ shows the probability of the query identity appearing in the top ranks of the candidate list. In this paper, we report the Rank-1 accuracy. mAP^35^ is used to measure model performance. All experiments follow standard evaluation protocols to ensure the reliability of the results.

#### Implementation details

The proposed method and all experiments were implemented on a single NVIDIA A100 GPU using the PyTorch framework. The baseline model adopts ResNet-50^36^ as the feature extractor, with the backbone parameters initialized using ImageNet pre-trained weights. Input images are resized to 3 × 288 × 144. During training, data augmentation is applied using random cropping with zero-padding, random horizontal flipping, and random erasing. For infrared images, they are duplicated and stacked to form 3-channel inputs. Feature enhancement is performed using grayscale augmentation. During training, a mini-batch sampling strategy is employed, where each batch randomly selects 8 different identities. For each selected identity, three types of image modalities, namely visible, grayscale, and infrared, are provided, with four image instances per modality. For optimization, the Adam optimizer is used with a base learning rate of 0.0065. Considering the different learning characteristics of each network module, a differentiated learning rate initialization strategy is applied: the Batch Normalization layers and classifier module use the full base learning rate of 0.0065, while the other network modules use one-tenth of that, i.e., 0.00065. A staged learning rate decay strategy is adopted during training: the learning rate is reduced to 10% of the initial value at the 20th epoch and further decreased to 1% at the 50th epoch. The entire training process lasts for 80 epochs to ensure full model convergence.

### Comparison with state-of-the-art methods

To evaluate the performance advantages of the MS-CF framework, we first compare it with the current state-of-the-art cross-modality ReID methods. These methods include multimodal fusion-based approaches (MSLNet^37^, DMiR^38^, MTMFE^13^, TMCC^39^, SIDA^40^, SHFL^24^, CM²GT^41^, ACD-Intra^42^), graph neural network and attention mechanism-based approaches (DCLNet^9^, G²DA^43^, DSCNet^44^, PMT^45^), feature alignment-based approaches (AGW^1^, NFS^46^, SFANet^47^, TMD^48^, DARD^49^, Dual-MLPs^50^), auxiliary information-based approaches (DDAG^51^, DART^52^, GECNet^53^, LCNL^54^, TMAL^55^, AGPI²^56^), and metric learning-based approaches (VSD^57^, FCMI^12^, CIIM^58^). Table 1 presents the performance comparison results of MS-CF on the SYSU-MM01 and RegDB datasets, evaluated using Rank-1 accuracy and mAP. The mean denotes the average performance of mAP and Rank-1 across the two datasets. These key metrics consistently demonstrate the overall superiority of MS-CF.

**Table 1:** Comparison of the MS-CF model with some of the most advanced methods on the SYSU-MM01 and RegDB datasets.

Methods	Venue	SYSU-MM01				RegDB				mean	
		All search		Indoor search		Visible-to-infrared		Infrared-to-visible			
		mAP	Rank-1	mAP	Rank-1	mAP	Rank-1	mAP	Rank-1	mAP	Rank-1
AGW	TPAMI2021	47.65	47.50	62.97	54.17	66.37	70.05	65.90	70.49	60.72	60.55
VSD	CVPR2021	58.80	60.02	72.98	66.05	71.60	73.20	70.10	71.80	68.37	67.77
MCLNet	ICCV2021	61.98	65.40	76.58	72.56	73.07	80.31	69.49	75.93	70.28	73.55
NFS	CVPR2021	55.45	56.91	69.79	62.79	72.10	80.54	69.79	77.95	66.78	69.55
DDAG	TIFS2022	53.02	54.75	67.98	61.02	63.46	69.34	61.80	68.06	61.57	63.29
DART	CVPR2022	66.30	68.70	78.20	72.50	75.70	83.60	73.80	82.00	73.50	76.70
DCLNet	ACM MM2022	65.18	70.79	76.80	73.51	74.30	81.20	70.60	78.00	71.72	75.88
GECNet	TCSVT2022	51.80	53.40	62.90	60.60	78.50	82.30	75.60	78.90	67.20	68.80
DmiR	TCSVT2022	49.29	50.54	62.49	53.92	69.97	75.79	68.22	73.93	60.49	63.55
G²DA	PR2023	60.73	63.94	76.01	71.06	65.90	71.72	63.88	69.50	66.63	69.06
MTMFE	JITIP2023	60.57	62.56	73.86	65.06	74.39	76.10	71.04	72.18	69.97	68.98
FCMI	MVA2023	57.30	60.50	71.40	66.10	73.60	79.00	73.70	78.80	69.00	71.10
SFANet	TNNLS2023	60.30	65.70	80.10	71.60	68.00	76.31	63.77	70.15	68.04	70.94
TMCC	MFA2023	58.52	60.66	70.95	65.22	77.42	85.35	75.82	83.65	70.68	73.72
SIDA	CVIU2023	64.19	68.36	77.49	73.28	75.07	81.73	72.60	79.71	72.34	75.77
DSCNet	TIFS2023	69.47	73.89	82.65	79.35	77.30	85.39	75.19	83.50	76.15	80.53
PMT	AAAI2023	64.98	67.53	76.52	71.66	76.55	84.83	75.13	84.16	73.30	77.05
SHFL	NSPP2024	63.82	65.92	79.98	75.91	77.48	85.60	74.59	82.81	73.97	77.56
LCNL	IJCV2024	68.00	70.20	80.30	76.20	78.70	85.60	76.90	84.00	75.98	79.00
TMD	TMM2024	63.96	68.81	74.52	76.31	81.19	87.04	77.92	83.54	74.40	78.83
DARD	TIFS2024	65.65	69.33	81.91	77.21	85.39	86.19	85.09	85.53	79.51	79.57
CIIM	TPAMI2024	56.14	57.22	69.77	61.68	70.96	76.54	69.66	74.81	66.63	67.56
ACD-Intra	TIFS2024	71.17	74.44	82.75	78.98	83.28	84.71	84.72	87.18	80.48	81.33
TMAL	ICME2024	73.96	70.58	82.70	76.17	85.04	82.65	83.67	80.94	81.34	77.59
Dual-MLPs	TMM2024	67.49	70.59	80.24	75.98	76.39	S85.34	75.16	83.88	74.82	78.95
AGPI²	TIFS2025	70.58	72.23	84.25	83.45	83.89	**89.03**	83.04	**87.91**	80.44	83.16
CM²GT	PR2025	63.50	69.79	76.63	73.41	77.79	86.72	77.51	86.47	73.86	79.10
MS-CF		**77.56**	**78.77**	**88.50**	**87.42**	**86.95**	85.31	**85.24**	84.18	**84.56**	**83.92**

The best results are shown in bold. The underline indicates the second-best performance.

On SYSU-MM01 (All-Search), compared with the current state-of-the-art method TMAL, MS-CF achieves improvements of 8.19% in Rank-1 accuracy and 3.6% in mAP. TMAL leverages the text modality for high-level semantic alignment but makes insufficient use of low-level local features. It also lacks multi-stage end-to-end optimization and has limited noise handling capability, which restricts global retrieval and feature stability. In contrast, MS-CF adopts multi-stage hybrid modality alignment combined with grayscale modality bridging and collaborative preprocessing to realize progressive optimization from single-modality to hybrid-modality. This design balances low-level details and high-level semantics, thereby delivering superior cross-modality recognition performance and retrieval robustness on SYSU-MM01.

On RegDB, compared with the state-of-the-art method DARD, MS-CF achieves improvements of 1.56% in the visible-to-infrared setting and 0.15% in the infrared-to-visible setting in terms of mAP, but exhibits slightly lower Rank-1 accuracy. DARD adopts dual adversarial representation disentanglement and feature separation mechanisms that enhance the robustness of modality-shared features while reinforcing modality-specific information through adversarial perturbations. Its optimization objective prioritizes extreme Top-1 matching, following a strongly discriminative but globally weak paradigm that tends to boost Rank-1 while sacrificing ranking consistency across long candidate lists. In contrast, MS-CF leverages grayscale modality bridging and multi-stage hybrid alignment to emphasize global feature stability and cross-modality consistency, thereby achieving significantly higher mAP (i.e., overall ranking performance) yet slightly lower Rank-1 accuracy. On RegDB, our method consistently demonstrates higher mAP yet lower Rank-1 accuracy in both visible-to-infrared and infrared-to-visible directions. This phenomenon primarily results from divergent optimization objectives. Some methods (e.g., DARD, DSCNet, AGPI²) emphasize Top-1 discriminative ability by employing adversarial perturbations, feature disentanglement, or strong discriminative losses to quickly identify the most probable match under extreme conditions, thus boosting Rank-1. In contrast, MS-CF prioritizes multi-stage cross-modality consistency and global ranking stability, suppressing cross-modality noise and enhancing the robustness of shared features through grayscale bridging and progressive alignment. While this optimization improves overall retrieval ranking (mAP), it may slightly compromise Rank-1. Moreover, RegDB constitutes a small-scale dataset with one-to-one cross-modality pairing, where Rank-1 is highly sensitive to the model’s discriminative ability on individual samples. Therefore, MS-CF can be regarded as a robustness-first paradigm that is particularly suitable for large-scale complex scenarios (e.g., SYSU-MM01), whereas on RegDB it manifests as higher mAP yet slightly lower Rank-1.

**Fig. 2:**
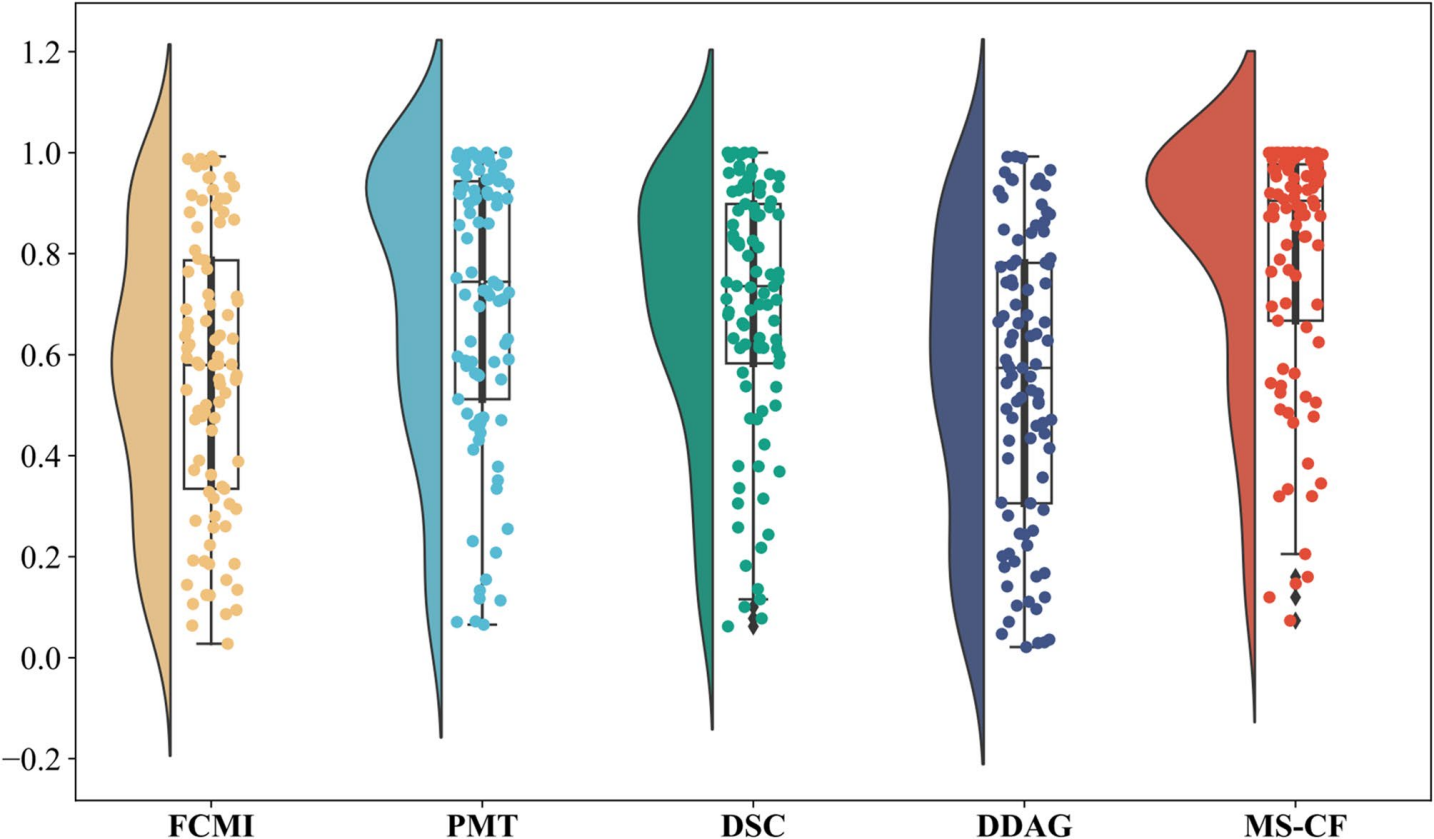
Comparison of different model results for AP value with MS-HMA.

We conducted a visual analysis of the feature alignment performance of the MS-CF framework, which incorporates the MS-HMA module. A raincloud plot was employed to comprehensively illustrate the model’s performance from multiple perspectives. As shown in Fig. 2, each color encodes a unique identity, and each dot corresponds to the AP (Average Precision) value calculated between a query image and a gallery image. Higher AP values indicate more accurate identity predictions. These results are obtained on the SYSU-MM01 dataset under the all-search experimental setting, where visible images are used to retrieve infrared images.

From Fig. 2, it can be observed that the score distributions of FCMI and DDAG exhibit a clear long-tail trend, indicating that many samples remain in low-confidence regions even after alignment. This volatility is closely related to the design of each method. Although FCMI incorporates class-center consistency and a modality-adversarial mechanism, its strategy focuses on coarse-grained feature constraints. It lacks modeling of structural details and intermediate semantic layers, resulting in significant local alignment deviations. DDAG is capable of local structural aggregation. However, it lacks an explicit staged modality alignment process and fusion control mechanism, making it difficult to achieve semantic consistency across modalities at a global level. As a result, the alignment performance varies greatly between samples.

In contrast, PMT leverages the grayscale modality to achieve staged modality transitions, effectively alleviating the explicit modality conflict between visible and infrared images. DSC introduces semantic consistency modeling at the channel dimension, improving alignment accuracy. However, as shown in the distribution plots, both methods still exhibit tail feature drift in the fused feature space, with a small number of samples scoring significantly lower. The underlying reasons are as follows. Although PMT employs a progressive alignment strategy, it lacks a consistency reconstruction and supervision mechanism for the fused shared features, and DSC’s channel-level consistency modeling fails to incorporate hierarchical semantic awareness, making it difficult to cover distribution discrepancies across different semantic levels, thereby leaving structural noise in the fusion process.

Compared to the above methods, our proposed MS-CF demonstrates superior alignment stability. As shown in Fig. 2, MS-CF not only achieves the highest median scores but also exhibits the smallest distribution dispersion. This demonstrates the MS-HMA module’s effectiveness in promoting cross-modality semantic consistency, mitigating intra-modality discrepancies, and reducing redundant information during feature fusion.

**Fig. 3:**
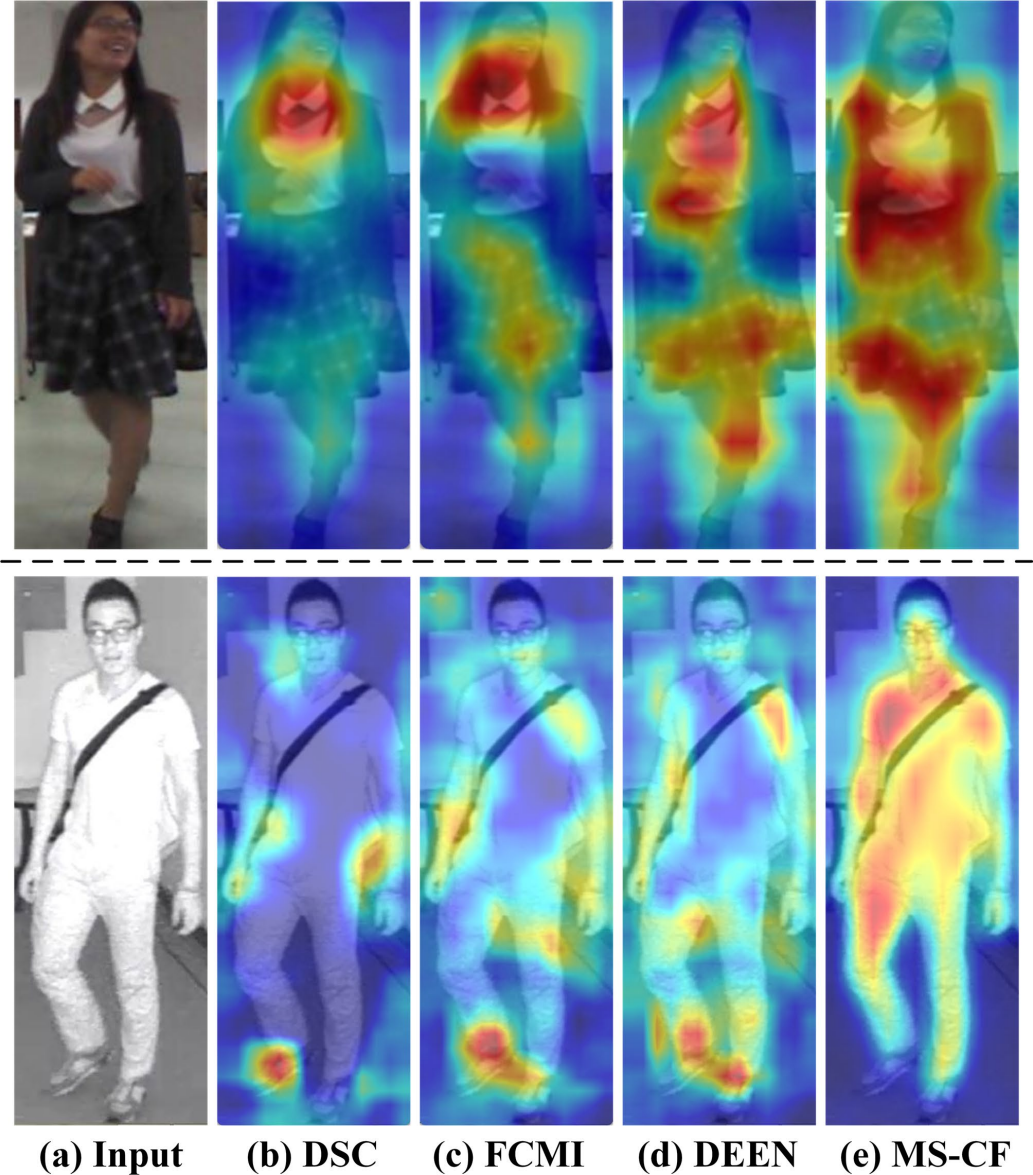
Comparison of Different Model Results for Heatmaps with DCA. We use Grad-Cam + + to visualize the results for the same person. (a) shows the input RGB and infrared images, (b) shows the corresponding heatmap generated by DSC, (c) shows the corresponding heatmap generated by FCMI, (d) shows the corresponding heatmap generated by DEEN, and (e) shows the heatmap generated by MS-CF after integrating DCA.

To further investigate the importance of feature information retention in VI-ReID, we applied the Grad-CAM technique^59^ to visualize the model’s attention regions in Fig. 3a, validating the effectiveness of DCA from the perspective of feature learning. As shown in Fig. 3e, MS-CF demonstrates more prominent attention responses in Grad-CAM visualization, effectively suppressing irrelevant information, particularly in edge regions. In contrast, DSC (Fig. 3b) exhibits a scattered attention distribution, leading to feature loss; FCMI (Fig. 3c) produces more concentrated attention but overly relies on local salient regions while neglecting global context; DEEN (Fig. 3d) alleviates modality discrepancies by generating different embeddings and achieves a relatively balanced attention distribution, but its discriminative capability remains limited and is still affected by noise. By integrating DCA, MS-CF generates clearer and more discriminative heatmap results.

**Fig. 4:**
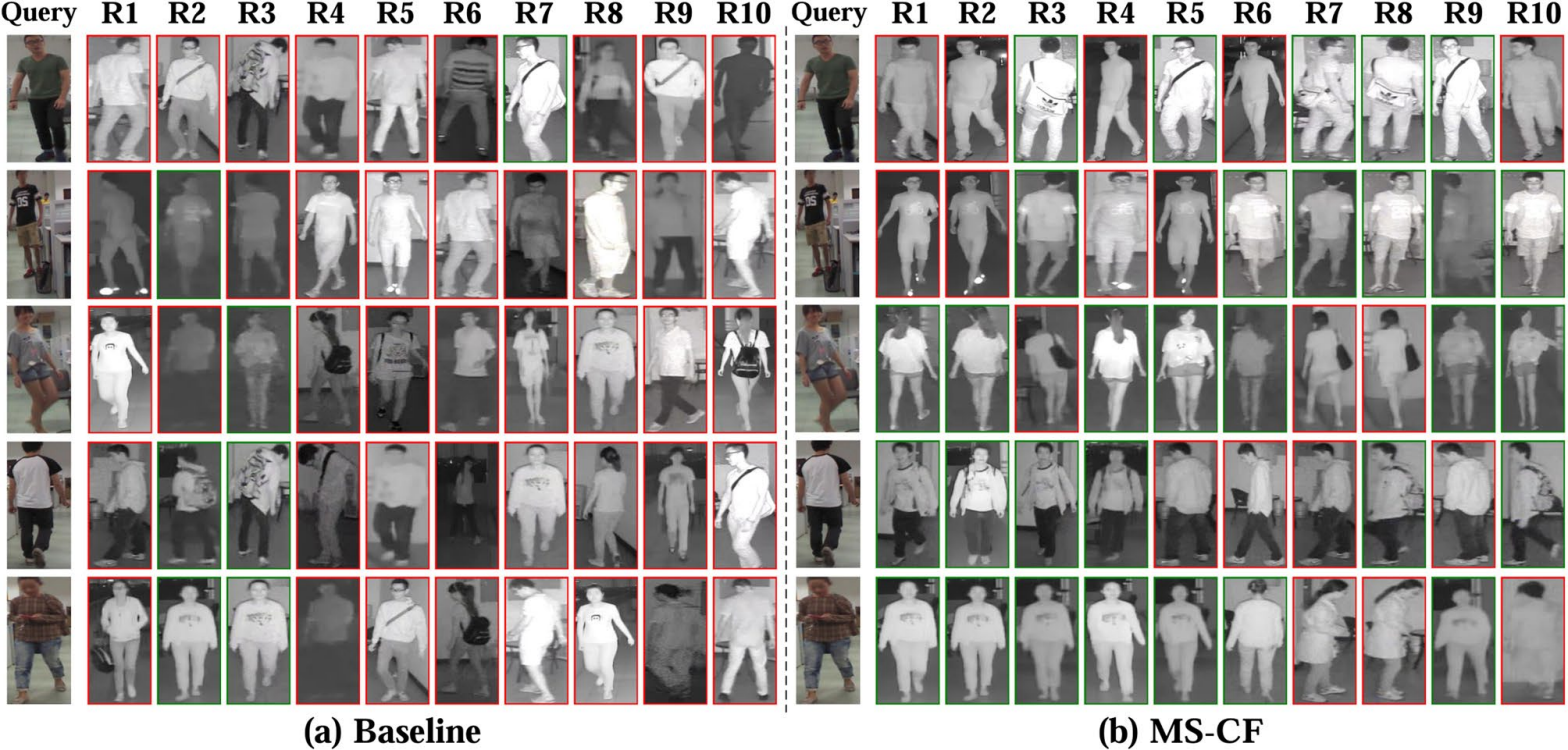
Comparison of Top-K retrieval results (RGB as query) between the baseline and MS-CF on the SYSU-MM01 dataset.

**Fig. 5:**
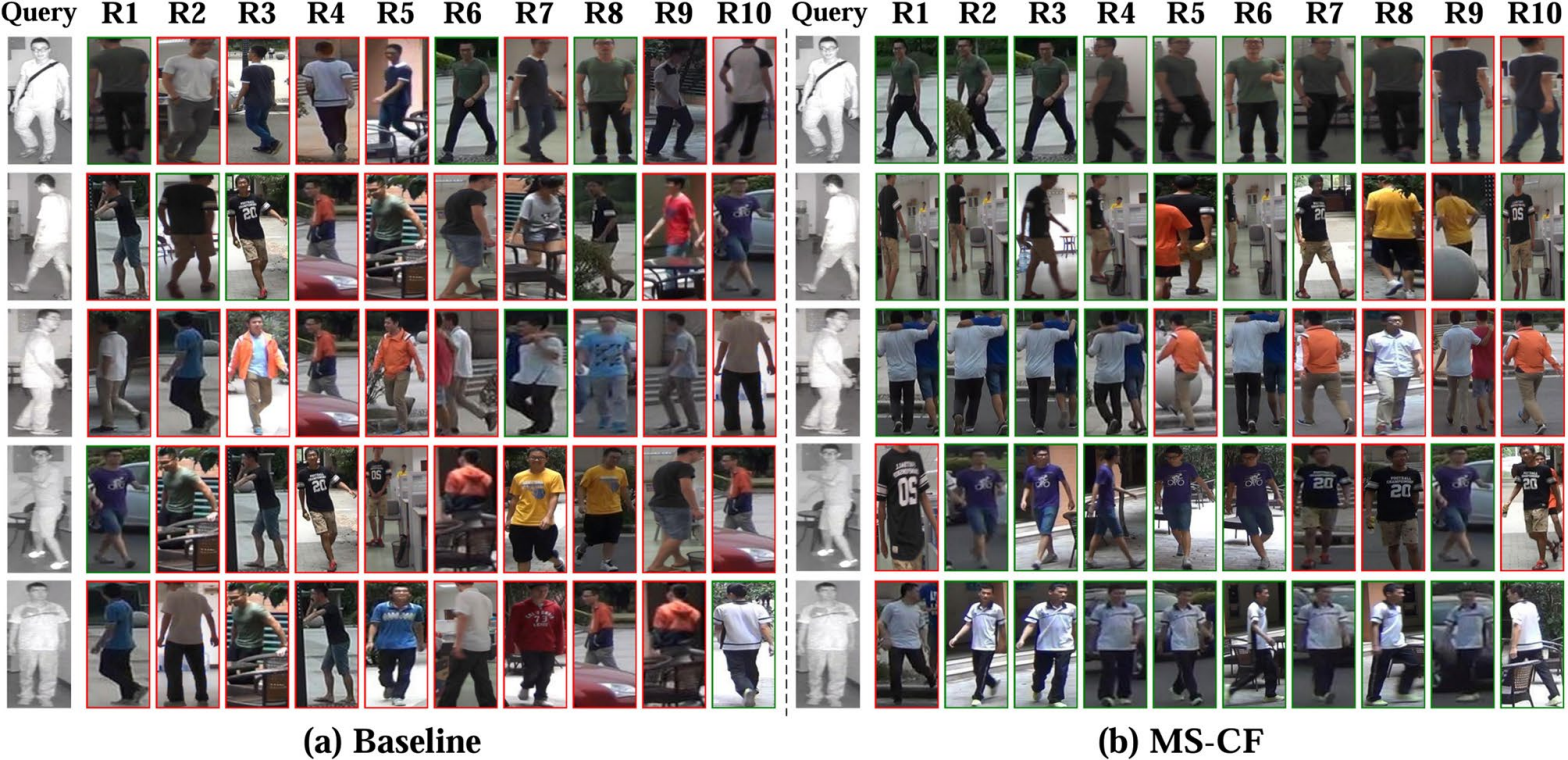
Comparison of Top-K retrieval results (IR as query) between the baseline and MS-CF on the SYSU-MM01 dataset.

To more intuitively demonstrate the retrieval performance of the model, Figs. 4 and 5 present the R1–R10 retrieval results with RGB and IR images as queries, respectively. It can be observed that correct matches are ranked higher and incorrect matches are significantly reduced, which verifies the effectiveness of the model in cross-modal matching.

**Fig. 6:**
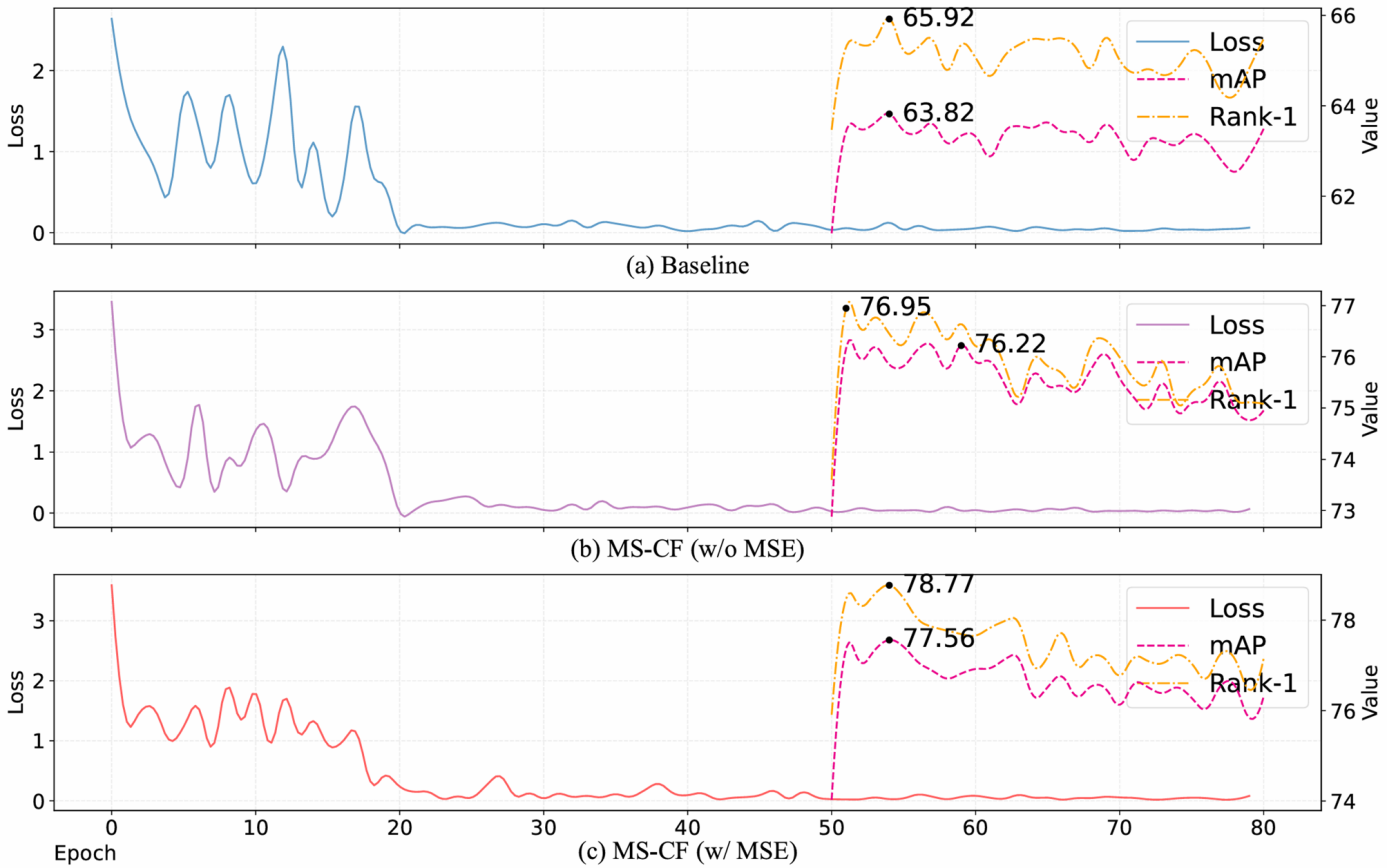
Comparison of the variations in loss, mAP, and Rank-1 between the baseline and MS-CF on the SYSU-MM01 dataset.

As shown in Fig. 6, all three methods exhibit a steadily decreasing loss during training, indicating effective model convergence. Compared with the baseline, MS-CF and its variants achieve faster convergence and lower training loss, demonstrating the effectiveness of the proposed method in optimizing feature learning. Further analysis shows that although MS-CF (w/o MSE) achieves lower training loss, its retrieval performance is not optimal, indicating that relying solely on the classification loss (CE) may lead to insufficient feature representation and limit cross-modal discriminability. In contrast, MS-CF with the MSE constraint achieves the best performance (mAP = 77.56, Rank-1 = 78.77) despite a slightly higher training loss. This demonstrates that the MSE constraint promotes cross-modal feature alignment, reduces modality discrepancies, decreases intra-class distances, and enhances inter-class separability.

### Ablation study

In this section, ablation studies are conducted on the SYSU-MM01 and RegDB datasets under the all-search and visible-to-infrared settings to systematically evaluate the impact of different configurations on model performance. MS-CF consists of three components, namely DCA, BFN, and MS-HMA, which do not operate independently but collaborate in a progressive manner. Specifically, DCA enhances feature representation, BFN normalizes feature distributions and alleviates modality discrepancies, and MS-HMA strengthens cross-modal semantic alignment. These components work together to achieve high-quality feature modeling and effective alignment. Experimental results show that their joint use yields the best performance, demonstrating a clear synergistic effect. Based on this, detailed ablation analyses of each component are presented below.

#### Comparison of integration results of different components

To evaluate the contribution of each module in the proposed MS-CF framework, we conducted ablation experiments by progressively introducing DCA, BFN, and MS-HMA based on the baseline model (B). The results in Table 2 show that incorporating DCA brings consistent performance improvements on both datasets. Compared with the baseline model (id1), on SYSU-MM01, the mAP and Rank-1 increase by 1.89% and 2.40%, respectively; on RegDB, they improve by 1.55% and 2.87%, respectively. These results indicate that integrating DCA into the backbone effectively enhances the cross-modal feature representation capability of the model, although the training time increases to approximately 2.31 times that of id1. Furthermore, experimental settings involving DCA (id5, id6, and id8) achieve further improvements in both mAP and Rank-1 while maintaining a moderate training overhead when combined with other modules. This can be attributed to the fact that DCA establishes cross-layer feature interactions, enabling effective fusion of shallow fine-grained textures and deep semantic information. Consequently, it preserves structural details while maintaining high-level semantic discriminability and improves cross-modal feature consistency through multi-level collaborative modeling.

**Table 2:** Evaluating the impact of different components on the MS-CF model.

Model					SYSU-MM01			RegDB			Mean		
idx	B	DCA	BFN	MS-HMA	mAP	Rank-1	Time(hour)	mAP	Rank-1	Time(hour)	mAP	Rank-1	
1	√				63.82	65.92	14.08	77.48	83.79	24.12	70.65	74.86	
2	√	√			65.71	68.32	32.59	79.03	**86.66**	24.28	72.37	77.49	
3	√		√		64.78	67.83	9.42	78.23	85.25	16.60	71.51	76.54	
4	√			√	68.25	66.76	8.83	84.83	84.39	16.80	76.54	75.58	
5	√	√	√		66.46	69.02	19.14	86.19	85.27	24.86	76.33	77.15	
6	√	√		√	69.75	67.68	19.16	86.47	86.22	25.48	78.11	76.95	
7	√		√	√	77.00	77.92	7.95	86.28	85.54	47.40	81.64	81.73	
8	√	√	√	√	**77.56**	**78.77**	**10.70**	**86.95**	86.49	**31.21**	**82.26**	**82.63**	

The best results are shown in bold.

Compared with id1, incorporating BFN further improves model performance, achieving 65.71% and 79.03% mAP on SYSU-MM01 and RegDB, respectively. In addition, the results of id5, id7, and id8 demonstrate that this module exhibits good compatibility and synergy with other components. BFN effectively alleviates the feature scale inconsistency caused by modality differences and improves training stability. Meanwhile, it also shows certain advantages in training efficiency. For example, in id7, the training time is reduced to approximately 0.56 times that of id1.

The MS-HMA module further enhances model performance, increasing mAP to 68.25% on SYSU-MM01 and 84.83% on RegDB. MS-HMA provides higher-quality feature representations through a progressive structure-level alignment mechanism. The results based on id4 (i.e., id6, id7, and id8) further verify the effectiveness of this module and show that it can improve computational efficiency to some extent. For instance, on SYSU-MM01, the training time of id7 is further reduced by approximately 10% compared with id4.

When the three modules are jointly employed (id8), the model achieves the best overall performance, reaching 77.56% and 86.95% mAP on SYSU-MM01 and RegDB, respectively, while maintaining a moderate computational cost. Moreover, considering the average results across the two datasets, this configuration also achieves the best overall performance, with 82.26% mAP and 82.63% Rank-1, demonstrating the complementarity and synergistic effects among the modules.

Overall, the individual and joint results of DCA, BFN, and MS-HMA indicate that MS-HMA is a key factor in improving model performance, while the combination of DCA and BFN extracts more fine-grained feature representations and provides an important foundation for the effectiveness of MS-HMA. These findings highlight the importance of considering the interactions among different components when designing a high-performance cross-modal recognition framework.

#### Effectiveness of different combinations of SEP and SPP

The DCA module consists of two components, SEP and SPP. Specifically, SEP models the global correlations between high-level and low-level features to enhance discriminative cross-modal representations, while SPP focuses on local details to capture spatially consistent structural information. To validate the individual contributions of each component and the rationality of their interactions, we conduct a series of ablation studies. The results indicate that different combinations significantly affect model performance (see Table 3), among which the parallel structure(SEP + SPP) achieves the best performance.

On the SYSU-MM01 dataset, SEP achieves slightly higher mAP and Rank-1 than SPP, suggesting that global semantic information plays a more critical role in this dataset. In contrast, on RegDB, SEP and SPP exhibit comparable performance, which may be attributed to the relatively simple feature distribution, where both global and local information are equally effective. Based on these observations, we further evaluate a sequential structure(SEP→SPP), where SEP first models channel-wise dependencies and SPP subsequently captures spatial dependencies. The results show that this structure achieves higher mAP than using SEP or SPP alone on SYSU-MM01, indicating their complementary nature, as SEP provides more discriminative input features for SPP. However, on RegDB, the mAP drops by 6.68%, suggesting that the sequential combination may disrupt the original global semantic representations.

Furthermore, introducing a dual SPP structure (SEP→SPP→SPP) does not improve performance on SYSU-MM01, indicating that the first SPP already captures the primary spatial dependencies, and repeated application may lead to redundancy or interference. In contrast, on RegDB, the dual SPP structure achieves the best performance. This is likely because the dataset exhibits relatively stable structures, while modality discrepancies mainly lie in the spatial domain. Stacking SPP modules can therefore further enhance spatial consistency.

Notably, in the parallel design, SEP and SPP process the input features independently, modeling channel-wise and spatial information simultaneously, and their outputs are subsequently fused. This strategy effectively avoids the information interference introduced by sequential structures and yields more comprehensive feature representations. On SYSU-MM01, this design achieves the highest mAP and Rank-1, demonstrating its effectiveness for multimodal feature fusion.

**Table 3:** Comparison of results of different combinations of SEP and SPP.

SEP/SPP	SYSU-MM01		RegDB	
	mAP	Rank-1	mAP	Rank-1
Only SEP	71.18	68.75	86.04	85.31
Only SPP	68.83	65.78	86.01	85.24
SEP→SPP	71.33	68.60	79.33	86.37
SEP→SPP→SPP	71.33	68.04	**86.95**	**86.49**
SEP + SPP	**71.49**	**68.99**	86.07	85.52

The best results are shown in bold.

#### Comparison of results with different SEP weight ratios

As shown in Table 3, the parallel combination of SEP and SPP outperforms each component used individually. The optimal performance varies across datasets. On SYSU-MM01, SEP plays a more significant role, whereas on RegDB, SPP becomes dominant. This indicates that their relative contributions depend on the modality characteristics and data distribution.

**Fig. 7:**
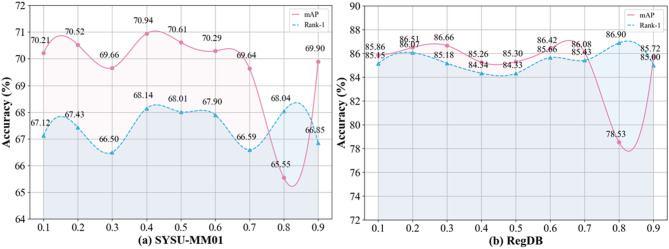
Comparison of results with different SEP weight ratios: (**a**) Results on the SYSU-MM01 dataset. (**b**) Results on the RegDB dataset.

On the SYSU-MM01 dataset (Fig. 7a), the model achieves the best performance when the weight of SEP is 0.4 (mAP = 70.94%, Rank-1 = 68.14%). This suggests that SEP, by combining convolution and self-attention mechanisms, effectively enhances global cross-modal feature alignment, particularly in capturing discriminative representations of key regions. Meanwhile, the local spatial modeling capability of SPP remains essential. When the SEP weight is excessively high (≥ 0.7), performance degrades significantly, indicating that a balance between the two components is necessary. Dominance of a single branch (either SEP or SPP) leads to performance deterioration. This phenomenon reflects that SEP and SPP exhibit nonlinear responses and complementary properties during feature extraction. Over-reliance on SEP weakens the contribution of low-dimensional spatial information, thereby impairing cross-modal alignment. In contrast, a proper balance enables the model to better integrate global semantic information and local spatial features, leading to improved robustness.

On the RegDB dataset (Fig. 7b), performance fluctuations are relatively small, indicating that the model is less sensitive to weight variations and remains stable. The best mAP (86.66%) is achieved when the SEP weight is 0.3, suggesting that SPP is more effective in capturing spatial and positional information. Its low-dimensional representation preserves spatial correlations while reducing computational complexity, making it particularly suitable for scenarios where modality discrepancies are mainly reflected in the spatial domain. Additionally, an extreme case is observed when the SEP weight is 0.8: the mAP drops to 78.53%, while the Rank-1 reaches its highest value (86.9%). This indicates that under this setting, global semantic consistency dominates, improving top-1 matching accuracy. However, insufficient modeling of local details leads to degraded overall ranking quality.

#### Comparison of results for different insertion positions of the DCA module

The DCA module introduces feature interactions and key region information from different stages of the backbone network. Since shallow layers mainly capture local details, while deeper layers encode higher-level semantic representations, we systematically investigate the impact of different insertion positions on model performance (see Table 4).

In the case of single-stage insertion, the performance gradually improves as the insertion depth increases, with block 3 achieving the best results. This is because DCA fundamentally serves as a semantic alignment mechanism, and deeper layers encode representations with stronger modality invariance. Therefore, inserting DCA at deeper stages leads to more effective cross-modal alignment and enhances discriminative capability. This observation further indicates that high-level semantic features are more beneficial for improving cross-modal discrimination.

For multi-stage insertion (e.g., block 1–3 or block 2–3), the performance is further improved, demonstrating the synergistic effect between convolutional features and attention-based representations. Specifically, we observe that different multi-stage insertion strategies yield comparable performance. This can be attributed to the fact that high-level features are already semantically consistent across modalities, which limits the room for further improvement through additional alignment. Moreover, since DCA acts as a feature refinement module rather than a dominant representation learner, performance variations across different configurations remain relatively small. Despite the small differences, inserting DCA at block 2 and block 3 (block 2–3) achieves the best performance on SYSU-MM01, with the highest mAP and Rank-1 (66.83% and 69.17%). This is because block 2 captures intermediate semantic representations suitable for coarse alignment, while block 3 encodes high-level semantics for fine-grained refinement. Such a design enables progressive cross-modal alignment, while avoiding noise from shallow layers and achieving an optimal balance. However, when DCA is inserted into too many layers (e.g., block 0–3), the performance slightly degrades. This is mainly due to feature redundancy and increased parameter overhead, which may negatively affect the optimization process. This phenomenon highlights the importance of carefully selecting appropriate insertion stages.

For the RegDB dataset, due to the one-to-one correspondence between image pairs, low- and mid-level features become more reliable for alignment. Therefore, introducing DCA at earlier stages helps effectively reduce modality discrepancies. As a result, multi-stage insertion at block 1–3 achieves the best performance. This finding indicates that the optimal insertion strategy of DCA should be adaptively adjusted according to dataset characteristics.

**Table 4:** Comparison of results for different insertion positions of the DCA module.

DCA	SYSU-MM01		RegDB	
	mAP	Rank-1	mAP	Rank-1
block 0	63.69	66.26	79.00	86.39
block 1	63.47	65.59	77.14	84.53
block 2	64.74	67.38	78.27	86.00
block 3	65.51	67.97	78.43	85.57
block 1–3	66.15	68.31	**79.52**	**86.67**
block 0–3	66.06	68.36	79.02	86.19
block 23	**66.83**	69.17	77.51	84.92
block 023	66.60	**69.46**	78.50	85.71

The best results are shown in bold.

#### Comparison of results of different combinations of L2 and ReLU

In this study, we introduce L2 regularization and the ReLU activation function after BN (Batch Normalization) to enhance the stability and representation capability of cross-modal features. BN alleviates feature distribution shifts, while ReLU improves the nonlinear representation capacity of the model. This combination has been widely adopted in deep neural networks. As shown in Table 5, using L2 alone outperforms using ReLU alone, indicating that feature normalization is more critical than pure nonlinear enhancement in cross-modal matching tasks. This is because L2 regularization effectively constrains the scale and distribution of features, thereby reducing modality discrepancies. In contrast, although ReLU can suppress negative responses, it lacks the ability to explicitly regulate feature distributions, resulting in inferior performance when used alone.

However, in visible-infrared person re-identification (VI-ReID), the significant distribution gap between modalities limits the effectiveness of the conventional BN and ReLU structure in feature alignment. To address this issue, we further impose L2 regularization on the scale and shift parameters of BN, which constrains feature magnitude and promotes cross-modal consistency.

Experimental results show that both L2 and ReLU individually bring performance improvements, while their combination achieves the best results. This demonstrates their complementary roles in enhancing feature alignment stability and discriminative capability. Although the performance gain is moderate, it remains consistent across datasets, indicating that the proposed normalization strategy effectively refines the learned feature representations.

**Table 5:** Comparison of results of different combinations of L2 and ReLU.

L2/ReLU	SYSU-MM01		RegDB		mean	
	mAP	Rank-1	mAP	Rank-1	mAP	Rank-1
Only L2	65.85	**68.31**	77.62	85.03	71.74	76.67
Only ReLU	65.07	68.04	77.48	83.79	71.28	75.92
L2 + ReLU	**66.10**	68.11	**78.23**	**85.25**	**72.17**	**76.68**

The best results are shown in bold.

#### Comparison of results with different MSE weight coefficients in MS-HMA

To maintain semantic consistency between aligned features obtained from different pathways, we introduce a consistency constraint based on MSE loss into the training objective. Unlike classification and metric learning losses that primarily focus on discriminative capability, the consistency loss directly regularizes the feature space and encourages modality-invariant representations. However, such a constraint may conflict with the objective of preserving discriminative features. Therefore, a weighting coefficient λ is introduced to balance the consistency loss and the primary task losses, as shown in Table 6. If the weight of this loss is not properly set, its relatively large gradients may dominate the training process and hinder the optimization of the main task. To address this issue, we use the coefficient λ to control its influence.

**Table 6:** Comparison of results with different MSE weight coefficients in MS-HMA.

MS-HMA/λ	SYSU-MM01		RegDB	
	mAP	Rank-1	mAP	Rank-1
0.1	77.03	77.61	**84.83**	**84.39**
0.3	**77.56**	**78.77**	84.21	83.41
0.5	77.00	77.44	83.60	83.00
0.7	76.91	77.66	82.95	81.88
0.9	76.82	77.35	82.09	81.14

The best results are shown in bold.

Experimental results on the SYSU-MM01 dataset show that the best performance is achieved when λ = 0.3, indicating that the consistency constraint at this level effectively stabilizes the cross-modality fusion process without suppressing the discriminative capability of modality-specific features. In contrast, the RegDB dataset, which has smaller modality discrepancies and stronger paired structure, exhibits optimal performance at λ = 0.1. This suggests that such data benefits more from preserving structural differences between modalities. We further conducted supplementary experiments on the SYSU-MM01 dataset with λ = 0.2 and 0.4. The results show marginally degraded performance compared to λ = 0.3 and exhibit noticeable instability across multiple training runs. We attribute this to the nonlinear coupling between the consistency loss and the primary task loss, which increases the model’s sensitivity to training data under small λ values, thereby hindering consistent performance improvements. To ensure clarity and reproducibility, we report only the representative λ values in the main paper.

## Conclusion

In this paper, we propose an efficient MS-CF model, which integrates three core components, namely DCA, BFN, and MS-HMA, to explore the semantic correlations between visible and infrared modalities in a multi-stage hierarchical manner. This approach achieves multi-dimensional fusion of discriminative information at different stages. The combination of DCA and BFN constructs a stable and discriminative feature space prior to feature alignment. We observe that single-stage modality alignment struggles to effectively bridge the modality gap in high-dimensional feature spaces, and semantic shifts further exacerbate the issue. Motivated by this, we introduce MS-HMA in the alignment stage to progressively map the initially aligned cross-modality features into a shared space. This achieves mixed-modality alignment without additional parameters. This design significantly enhances the model’s representational capacity in complex scenarios. Extensive experiments conducted on two widely used datasets, SYSU-MM01 and RegDB, demonstrate the superior performance of MS-CF and its components. Compared with state-of-the-art methods, our approach achieves notable improvements in recognition accuracy, stability, and generalization capability.

Although the MS-CF framework achieves promising performance in visible-infrared person re-identification, it still has several limitations that require further investigation. First, in terms of computational complexity, the current Dual-path Cross-layer Attention models features from different dimensions through two attention operations, which leads to a relatively complex architecture. Moreover, it lacks the ability to dynamically adjust the weights or importance of the two attention branches, which may limit its adaptability in complex environments. In addition, when applied under strong domain shifts, the multi-stage feature alignment strategy, although improving cross-domain robustness to some extent, may still suffer from performance degradation. Furthermore, in real-world scenarios, when targets are partially occluded, global feature-based matching methods are prone to failure. To address these limitations, future work can be explored in the following directions: (1) designing lightweight networks to adaptively predict attention fusion weights; (2) introducing domain generalization or adaptive learning strategies to enhance robustness; and (3) incorporating local consistency constraints to identify occluded regions. Overall, the proposed multi-stage feature alignment method demonstrates good extensibility and provides valuable insights and a solid foundation for cross-modal person re-identification and future multimodal vision research.

## Data Availability

The code in this study are available on request from the corresponding author. The direct links to the datasets used in this study are provided below. SYSU-MM01: https://gitcode.com/Universal-Tool/d4974, RegDB: https://gitcode.com/Premium-Resources/47a2b.
